# Supplementary material for: Climate change projected to impact structural hillslope connectivity at the global scale
Source: Nat Commun. 2023 Oct 25;14:6788. doi: 10.1038/s41467-023-42384-2 (PMC10600250; doi:10.1038/s41467-023-42384-2)
Supplement: Supplementary file 1 — Supplementary Information [file 41467_2023_42384_MOESM1_ESM.pdf]

Supplemental Information for

Climate change projected to impact structural hillslope connectivity at the global scale

Alexander T. Michalek[1], Gabriele Villarini[1,2*], and Admin Husic[3]

[1]Department of Civil and Environmental Engineering, Princeton University, Princeton, NJ, USA.

[2]High Meadows Environmental Institute, Princeton University, Princeton, NJ, USA.

[3]Department of Civil, Architectural, and Environmental Engineering, The University of Kansas, Lawrence, KS, USA.

*Corresponding author: Gabriele Villarini, gvillari@princeton.edu

Supplementary Figures

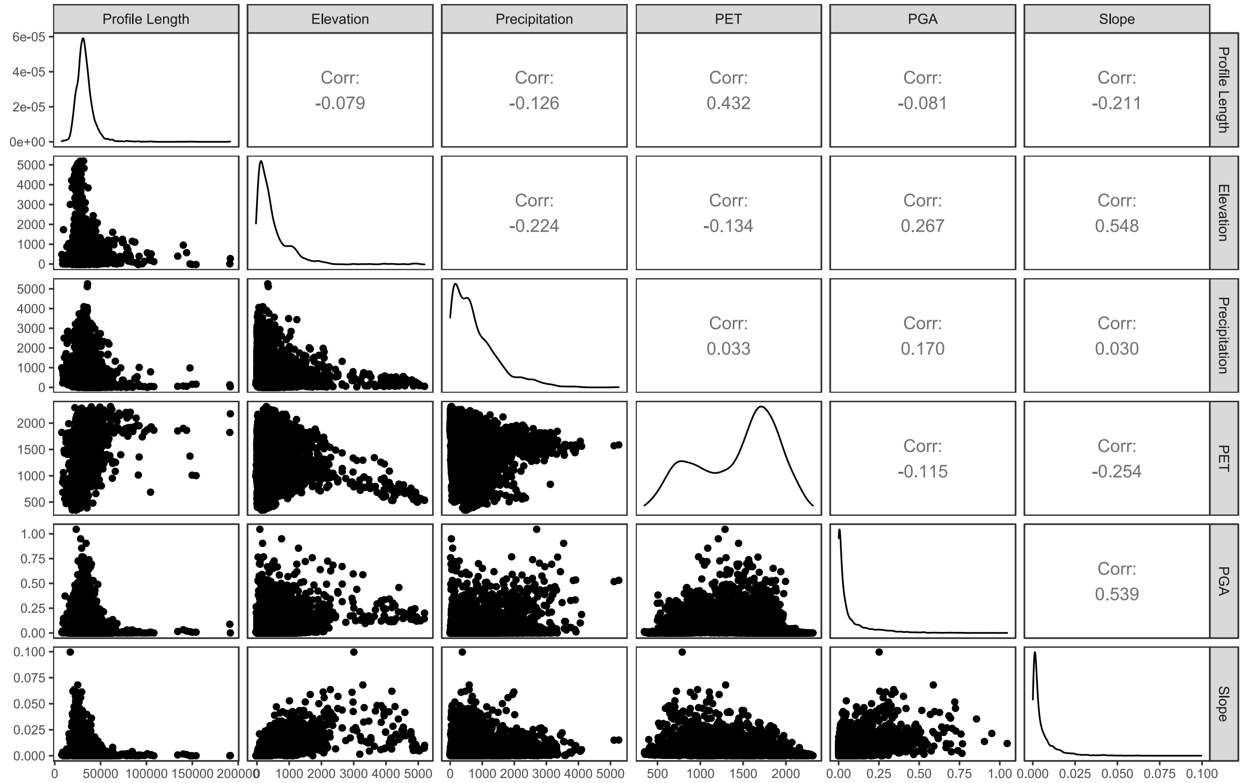

Figure S1. Correlogram of model predictors at HydroSHEDs level 5 basin size. Predictor values are from Ref[1].

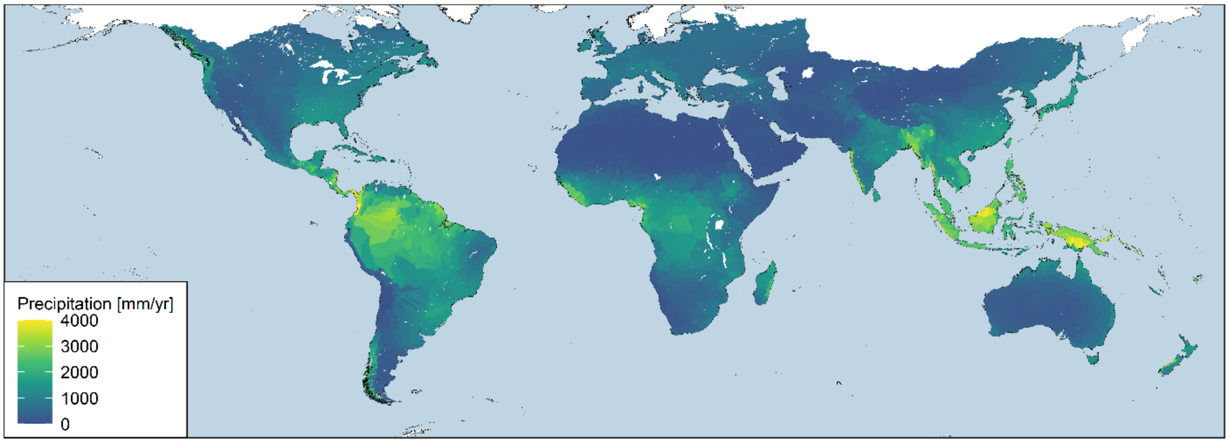

Figure S2. Historical average annual precipitation. Data for the period of 1970 to 2000 based on WorldClim dataset[2].

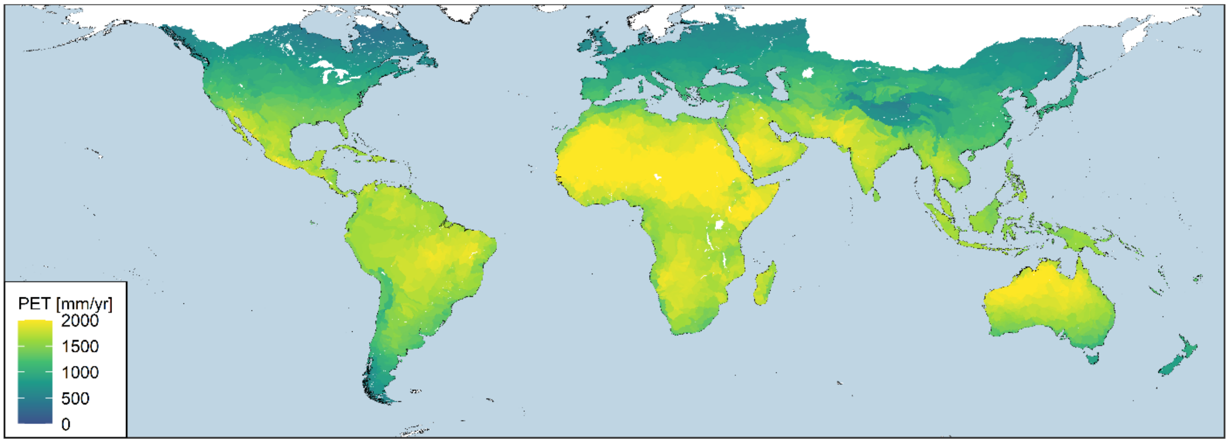

Figure S3. Historical average annual potential evapotranspiration (PET). Data for the period of 1970 to 2000 is based on WorldClim dataset[2].

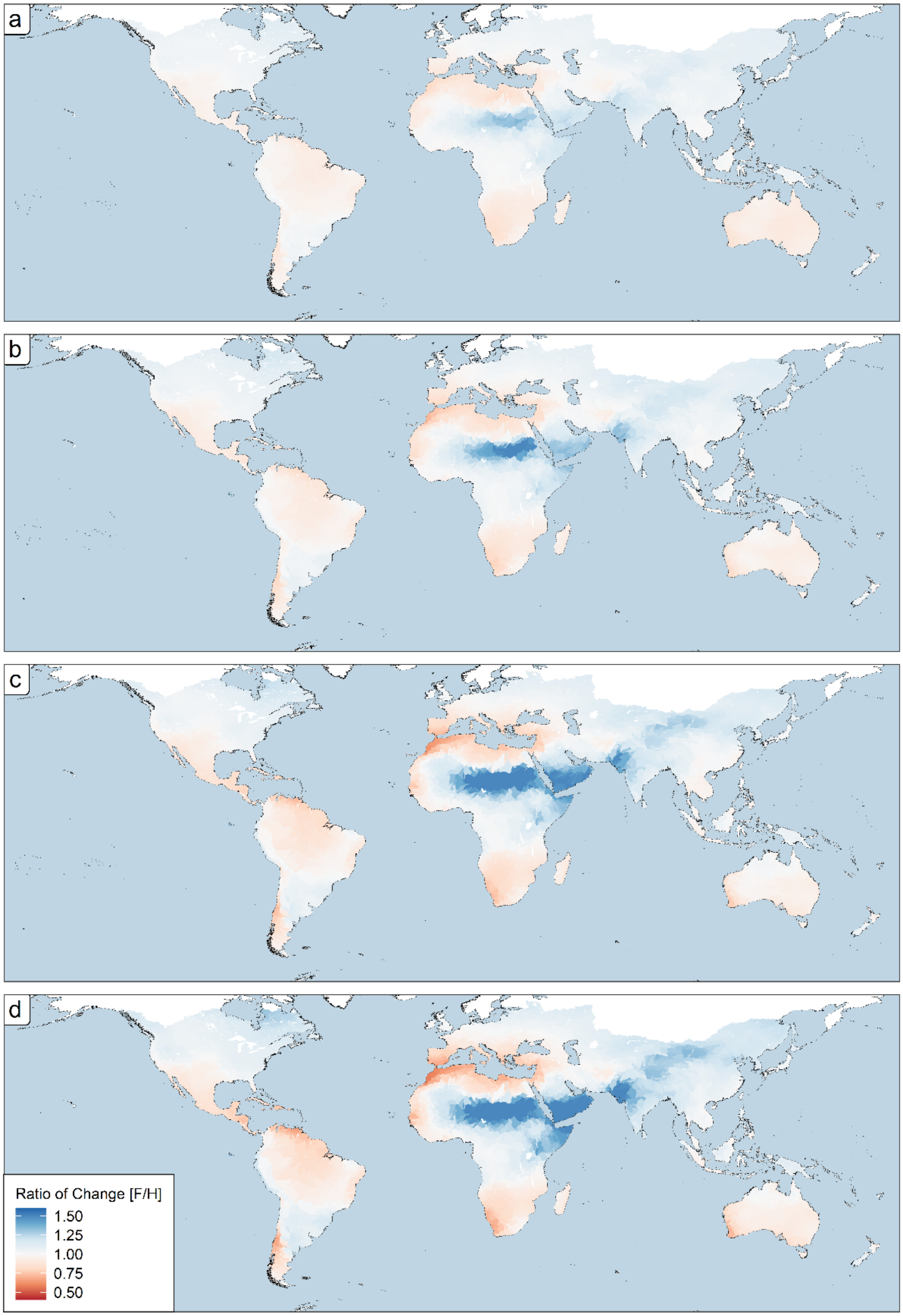

Figure S4. Ratio of future (2070 to 2100) to historical (1970 to 2000) annual average precipitation. The ratio is based on 34 climate model outputs from the Coupled Model Intercomparison Project 6 (CMIP6)[3] utilizing the Shared Socioeconomic Pathways (SSPs) of a) SSP126, b) SSP2452, c) SSP370, and d) SSP585.

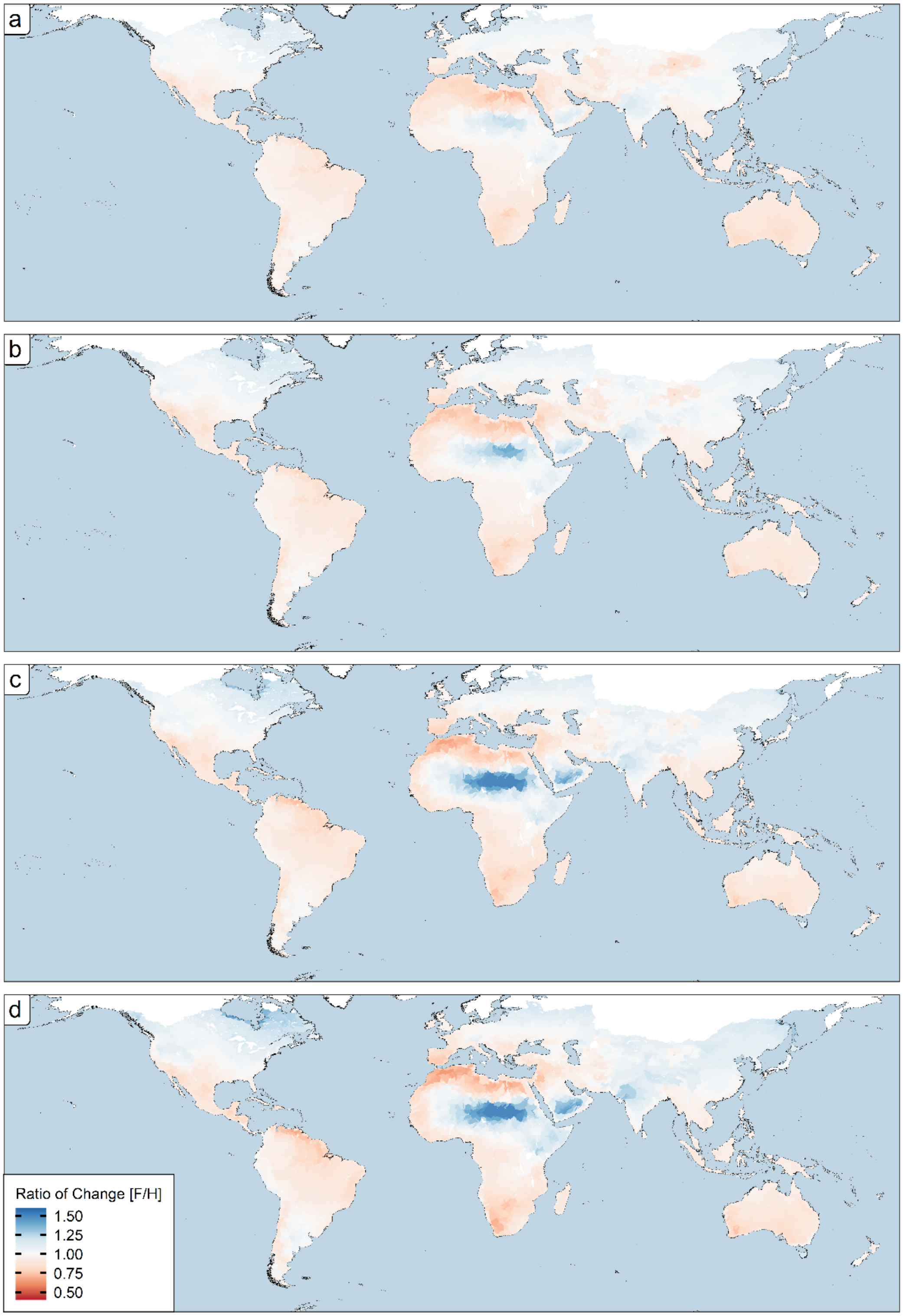

Figure S5. Ratio of future (2070 to 2100) to historical (1970 to 2000) annual average potential evapotranspiration. The ratio based on 34 climate model outputs from the Coupled Model Intercomparison Project 6 (CMIP6)[3] utilizing the Shared Socioeconomic Pathways (SSPs) of a) SSP126, b) SSP2452, c) SSP370, and d) SSP585.

Supplementary Tables

Table S1. List of distributions utilized in model development. The distributions are for use with the Generalized Additive Models for Location, Scale and Shape (GAMLSS).

Number of Parameters	Distributions
2	Normal, Logistic, Gumbel, Reverse Gumbel
3	Power Exponential family, t-family, Skew Normal family
4	Generalized-t, Normal exponential t, Skew Power exponential family, Sinh-Arcsinh family, Skew t family

Supplementary References

1. Seybold, H., Berghuijs, W. R., Prancevic, J. P. & Kirchner, J. W. Global dominance of tectonics over climate in shaping river longitudinal profiles. *Nat. Geosci.* **14**, 503–507 (2021).

2. Fick, S. E. & Hijmans, R. J. WorldClim 2: new 1-km spatial resolution climate surfaces for global land areas. *Int. J. Climatol.* **37**, 4302–4315 (2017).

3. Eyring, V. *et al.* Overview of the Coupled Model Intercomparison Project Phase 6 (CMIP6) experimental design and organization. *Geosci. Model Dev.* **9**, 1937–1958 (2016).
